# The Nerve Element in Surgical Pathology

**Published:** 1897-09

**Authors:** J. McFadden Gaston

**Affiliations:** Atlanta, Ga.


### Selections.

### THE NERVE ELEMENT IN SURGICAL PATHOLOGY.

By J. McFADDEN GASTON, M. D., Atlanta, Ga.

THE interlacing of the nerves with the different structures of the body gives energy to every vital organ in health and aggravates their disorder in disease.

Neuralgia in all its protean forms is not a mere functional derangement of the nerve centers, but depends in most cases upon local modifications resulting from inflammatory action in the neurilemma or parenchymatous structure of the affected nerve. It may also exist in a spurious form connected with compression upon the trunk or from cicatricial adhesions after operations. The impression that contraction of the nerves gives rise to painful development has led to nerve stretching, but little advantage has attended this procedure, thus showing that nerve shortening is not pathognomonic.

Rheumatic complications involving various regions of the body are dependent upon the nerve supply to the part and the acute sensibilities of such structures renders anodynes of the greatest importance in the treatment of such disorders. Inflammatory processes are accompanied by pain from the entrance of sensory branches of the nerves into the organs and the means adopted for the relief of inflammation must include the control of the neurotic disturbances by combating pain. It is fair to infer that all agencies for the mitigation of pain have a curative effect upon the structures involved in disease.

The reciprocal influéence of body and mind in the progress of most physical disorders depends upon their connection through the nervous system.

Our deficient knowledge of the etiological factor involved in shock inclines the speaker to the view that a continuous baleful influence is propagated to the nerve centers from the disintegration of the structure involved and that this may be modified by amputation with a clean incision through sound tissues above the point of injury, soon after such violence to the parts. The injury inflicted upon the superficial cutaneous nerve fibrils is extended to the internal organs by the correlation of nerves and capillaries with the viscera. The notable effects observed from cups, sinapisms ànd blisters upon the skin are due to the sympathies established through this channel of the nerves. The eruption of teeth in children is attended by neurotic disturbances of a marked character. The various manifestations of the intimate relations of the nerves with the different structures of the body open the way for comprehending the rôle of the nerve element in surgical pathology.

The development of traumatic neuritis is the most common complication of surgical cases, and there is rarely any extensive lesion which is not accompanied by more or less pain, dependent upon inflammation of the nerve or its neurilemma. The irritation of the peripheral branch of a nerve may set up a train of disorders terminating in tetanus or lymphangitis, and the serious consequences of these affections are notorious. In the latter condition ganglions as well as lymphatics become involved in the inflammatory process and suppuration is set up in the course of the lymphatics, with the characteristic features of pyemia.

The subcutaneous use of simple distilled water has been resorted to with apparent effect upon the nervous system, and this proves a delicate response of the nerves to the action of agents introduced hypodermatically. The transmission of cutaneous modifications to the internal organs becomes, in numerous instances, simply the expression or delivery of a dynamic influence which operates through the nerves. Instead of local irritation there is a general influence upon the organism corresponding to the special property of the agent employed, and we must attribute the effect to the conduction of medicinal powers from the point of introduction through the various channels of communication with the dependent structures.

The chief features in the relations of the nervous system to other structures involved in surgery lead to the following deductions:

1. The cutaneous development of the minute branches of the cerebro-spinal system of nerves, and the ganglionic ramifications of the great sympathetic, are so related to the capillaries as to establish a reciprocal action and reaction between them and the great nerve centers.

2. The vaso-motor nerves are so intimately linked with the excito-motor and excito-dynamic system of nerves that impressions made through the superficial afferent nerves are conveyed to all the corporeal structures and tissues, so as to produce their effects upon different organs.

3. Reflex phenomena depend upon a complex interchange of local pathological conditions with the nervous ramifications to remote parts of the body.

4. The fountain-head of energy for all the functions lies in the nerve centers and by controlling emanations from this source, the vital forces will be propagated with regularity and uniformity to all the remote parts of the physical organisation. On the contrary, a hurtful influence disseminated from the nerve centers entails disease upon the different organs.

5. The means to be adopted for averting injurious impressions upon the nerve centers, and the measures to be used for the correction of their derangement, make up the whole prophylactic agency of hygiene and include all the therapeutic appliances in the treatment of diseases, as well as the application of surgical measures.

6. Close observation of the various modifications of the nerve element on the physical organism should reveal its direct influence in surgical pathology and lead the surgeon to the adoption of proper means of relief.—*Southern Medical Record.*

